# Why is Mental Health Care Necessary During Hospitalization?

**DOI:** 10.3389/ijph.2022.1605153

**Published:** 2022-11-01

**Authors:** Érika Yánez

**Affiliations:** ^1^ University of Salamanca, Salamanca, Spain; ^2^ Pontificia Universidad Católica del Ecuador, Quito, Ecuador

**Keywords:** mental health, integrated care, public healh, mental health and wellbeing, hospitalization

**The IJPH series “Young Researcher Editorial” is a training project of the Swiss School of Public Health.**

Seriously ill or injured patients may require hospitalization to restore their physical health, but admission to hospital may have reduce the psychological and emotional well-being of patients and threaten their social stability. To understand health consequences beyond physical well-being, we must better understand the effect of hospital stays on patient mental health. In Latin America, patients with chronic disease are more likely to suffer psychological distress than patients with acute disease. Chronic disease patients may be lonely and socially isolated [1], and have a higher incidence of substance use than general population [2]. When patients suffer from both physical ailments and mental health problems, they are more likely to have prolonged hospital stays [3].

Researchers in Latin American have sought to identify associations and factors that predict the risk hospitalized patients will develop mental health problems. A study in Paraguay found that factors such as diagnosis, cause of hospitalization, prognosis and length of stay may predict the likelihood that patients will develop anxiety disorders and depression after prolonged hospitalization [4]. And a study at a Burn Unit in Baranquilla, Colombia found that a long hospital stay is associated with anxious-depressive states, lower patient’s capacity for emotional expression and adaptation disorders [5]. Both of these studies suggest that patients would benefit from mental health care interventions while they are in hospital.

Though non-pharmacological mental health care effectively reduces anxiety, distress, and pain, improves self-esteem and coping capacity, and increases the likelihood people will accept treatment, spend less time in the hospital, and even need less anesthesia and fewer analgesics [6], can Latin American health care systems provide it? Mental health disorders account for over a third of total disabilities in the Americas and current investment is too low to effectively reduce the public health burden of these diseases [7]. In Latin America, this has sometimes created large disparities in mental health care: those occupying the lowest socioeconomic strata have least access to care because patients must spend money out-of-pocket to use private services [8]. In these systems, mental health is not considered a human right that should be available to all, regardless of their socioeconomic status.

Provision of mental health care is already limited in health care systems based on the traditional medical-biological model of care. A 2014 study in Ecuador found that only 1.46% of the total budget for public health was spent on mental health care, and more than half that funding went to psychiatric hospitals, stripping resources from the first and second levels of care [9]. The World Health Organization’s Comprehensive Plan of Action makes clear that there is a severe shortage of specialized and general mental health professionals: almost half of the world’s population lives in countries where there is one or less psychiatrist for every 200,000 or more people. Mental health care providers trained to use psychosocial interventions are even scarcer [10].

The right to health means we should all have access to the health services we need, when and where we need them, without posing undue financial burdens. The literature makes clear that mental health care during hospitalization can reduce the time and use of medical and hospital resources and improve patients’ physical and mental health, so reducing the chance patients will develop psychological or psychiatric comorbidities during hospitalization should be a priority public health investment.
